# Drug overdose deaths during prison riots and mental states of prisoners: a case study

**DOI:** 10.3389/fpsyt.2024.1377995

**Published:** 2024-09-13

**Authors:** Luca Tomassini, Gianni Giuli, Edoardo Bottoni, Maria Chiara David, Roberto Scendoni

**Affiliations:** ^1^ International School of Advanced Studies, University of Camerino, Camerino, Italy; ^2^ Department of Pathological Addictions, Territorial Health Authority of Macerata, Macerata, Italy; ^3^ Department of Anatomical, Histological, Forensic Medicine and Orthopaedic Sciences, Section of Legal Medicine, Sapienza University of Rome, Rome, Italy; ^4^ Department of Public Security, Central Health Directorate, Forensic Toxicology Research and Laboratory Center, Ministry of the Interior, Rome, Italy; ^5^ Department of Law, Institute of Legal Medicine, University of Macerata, Macerata, Italy

**Keywords:** prison riots, suicide, prison environment, recreational drug use, overdose, crowd effect, impulse control disorders

## Abstract

Prison riots, though often sensationalized in the media, have profound consequences, with a significant death toll. Prison populations, historically plagued by psychiatric disorders, witness high rates of suicide, particularly linked to turbulent events like riots. This study examines three drug overdose deaths resulting from a prison riot during the initial wave of the SARS-CoV-2 pandemic in Italy. To ascertain the nature of these deaths, a comprehensive toxicological analysis was conducted. Immunochemical screening and gas chromatography-mass spectrometry were employed to detect a spectrum of drugs, including MDMA, methadone, morphine, cannabis derivatives, benzodiazepines, and others. The toxicological findings revealed high concentrations of various substances in the biological fluids of the deceased inmates. Tramadol and mirtazapine were implicated in one case, while methadone was a common factor in the deaths of two inmates, one of whom also ingested diazepam. The synergistic effects of substances were explored, with methadone identified as a leading cause of death in two cases. Prison riots exacerbate drug abuse issues within prisons, leading to mass intoxication and overdose, as witnessed in historic incidents globally. The study underscores the challenges in determining whether such deaths are accidental, intentional (suicidal), or a consequence of uncontrollable drug consumption during a riot. The prison environment also amplifies pre-existing psychiatric disorders, and incidents like riots can trigger a cascade of uncontrollable psychological reactions. The three potential scenarios are drug dependence, accidental overdose in recreational drug use, and suicide attempts through substance ingestion.

## 1 Introduction

Prison riots are particularly dramatic events that are covered on local or national news but receive less attention as a phenomenon of scientific research. Nonetheless, one of the direct consequences of such riots is a consistent death toll with multiple causes (1). From a historic and scientific perspective, prison populations are notoriously composed of subjects affected by one or more psychiatric disorders, ranging from substance abuse to depressive disorders, with high rates of suicide (2–4). Indeed, the resulting self-injurious behavior often makes it difficult to determine whether or not a death under these circumstances was intentional (5).

During the first wave of the SARS-CoV-2 pandemic in Italy, the first Western country to face the viral outbreak in 2020, a series of prison riots took place after some government decrees temporarily revoked some of the detainees’ rights, including visiting hours, while reinforcing security measures. In a central Italian detention facility, a riot occurred and national news reported that a total of fourteen detainees were injured; eight required hospitalization, three were admitted to intensive care, and three died after raiding the infirmary and taking drugs that had been kept there (6–8).

As reported in the document “Prison and drugs in Europe: current and future challenges” of 2021, it is noteworthy that individuals in prison exhibit higher rates of drug use and related issues compared to the general population (9). Despite being prohibited, illicit substances are prevalent within detention facilities. In fact, the prevalence of drug use among incarcerated individuals generally remains higher compared to the general population in the community. Studies conducted between 2004 and 2013 suggest that in Europe, between 20% and 45% of individuals with incarceration experience have used drugs while in prison (9, 10). During detention, patterns of drug use may also change as individuals adapt to the prison environment. People who use drugs may resort to using new substances when their drug of choice is not available in prison, or they may transition to a substance that is more readily accessible within the prison setting (9, 11).

Nevertheless, the misuse, or non-medical use, of prescription medications refers to the intentional use of prescribed drugs outside of their intended indication or the use of prescription drugs obtained illegally (12). The prescription drugs with the highest potential for abuse, not only in prison, are opioids, benzodiazepines, Z-drugs, and gabapentinoids (12–14). In this regard, prisons have been identified as high-risk environments for the misuse and associated harm of prescription drugs as well (15).

Although prisons provide a more human and ethical alternative to corporal and capital executions, they have been the scene of riots and disorder ever since they were founded (16). Perhaps the most emblematic cases to have been investigated by the scientific community are the Attica Prison Uprising of 1971 and the New Mexico State Penitentiary riot near Santa Fe in 1980 [United States] (1, 17). During the latter, 33 detainees died and 400 prisoners and staff were injured; half of the prison population needed hospitalization because of intoxication or overdose due to drugs stolen from the infirmary of the prison (17, 18). Similar episodes have been reported in national and local news around the world over the years: for instance, in 2014, during a riot at Uribana prison (Venezuela), 35 deaths were recorded after detainees raided the prison infirmary and overdosed on stolen drugs, and more than 100 prisoners needed hospitalization for drug intoxication (19, 20).

Two lethal mechanisms could be theorized in the cases under study: death due to overdose could have been accidental, or death could have been intended as the culmination of self-harming/suicidal behavior. Indeed, beyond the peculiarities of imprisonment itself [i.e. confinement, restriction of freedom, forced cohabitation], prison populations are known to be characterized by drug abuse issues: in Italy, the most recent data estimate that more than 25% of detainees are affected by some form of drug addiction (21), in line with data from other Western countries where, in the last decades, a distinct spread of drug abuse—including cannabis, heroine, and cocaine—has been recorded (22). The phenomenon is indeed widespread, regardless of the obvious issues in obtaining illegal substances from the external world (11, 23, 24).

In the examined cases, certain individuals, prior to instances of aggression, had ingested substances such as methadone, mirtazapine, benzodiazepines, or tramadol. The collective enthusiasm during the riot may have exacerbated impulse control disorders, particularly in those with a history of substance dependence, even in individuals without a positive history of substance use disorders, as in the cases presented (17, 25). Instead, interpreting the phenomenon from a neurobiological perspective, it is noted how the DSM-5 underscores the persistent alteration of cerebral circuits in substance use disorders, heightening the risk of relapse and intensifying the craving for substance consumption (26).

The aim of the study is to describe three cases of overdose occurring during a prison riot following an assault on the prison infirmary.

## 2 Description of cases

In 2020, in a detention facility in Italy, a prison riot took place in three different wards. Some 80 detainees occupied the prison and raided the infirmary, stealing drugs that were stored inside. Four detainees were hospitalized the same night for drug intoxication. At dawn the following day, once the riot had been quelled, one of the prisoners told prison guards that his cellmate seemed dead. The physician on duty arrived on site and confirmed the man’s death (Case #1). Shortly after, during physical health checks, a second (Case #2) and a third (Case #3) detainee were found lifeless in their beds and declared dead. In all three cases, no psychiatric disorders were reported. The bodies were subsequently subjected to complete autopsy. Potentially identifiable data, such as age, weight, and height, will be presented as ranges.

### 2.1 Toxicological analysis

In the cases under consideration, peripheral blood was collected from the inferior vena cava and placed into a container containing sodium fluoride. The collection was performed 155 hours postmortem for case 1, 149 hours postmortem for case 2-, and 152-hours postmortem for case 3. Samples were stored in a refrigerated environment at a controlled temperature of -20°C in a locked refrigerator. Further pathological examinations were conducted through subsequent blood draws.

In the context of toxicological assessments, a comprehensive screening analysis was conducted on urine samples using immunochemical techniques. The Multiline Drug Test^®^ was used for the qualitative detection of various substances such as MDMA, methadone, morphine, methamphetamine, cocaine, THC (cannabis derivatives), oxycodone, benzodiazepines, synthetic cannabinoids (JWH-018 and -022), ketamine, tramadol, and buprenorphine.

A general search for non-volatile organic compounds in biological samples was performed using gas chromatography-mass spectrometry (GC-MS). The samples underwent extraction with ethyl acetate in different environments (acidic, neutral, alkaline), and solid-phase extraction columns were employed. GC-MS analysis conditions included an Agilent 7820A Gas Chromatograph with 5975 Mass Detector and HP-5MS capillary column. Derivatization with BSTFA was the final step, to test for substances not otherwise detectable.

For the confirmation and quantification of methadone, a targeted search was conducted on central and peripheral blood samples, urine, and gastric contents. Deuterated internal standards were used, and liquid-liquid extraction was performed at pH 10–12. GC-MS analysis targeted specific ions [SIM], with quantitative assessment achieved by comparing peak areas of the analyte to deuterated internal standards.

Tramadol detection involved solid-phase extraction of peripheral blood and urine samples, with or without enzymatic hydrolysis. GC-MS analysis targeting specific ions (SIM) of tramadol and a deuterated internal standard (tramadol-d3) facilitated quantitative analysis.

Similarly, a targeted search for the active ingredients of benzodiazepines was conducted on peripheral blood and urine samples. Deuterated internal standards were used, and solid-phase extraction was performed, with or without enzymatic hydrolysis. GC-MS analysis targeted specific ions (SIM) of various benzodiazepines for quantitative assessment.

For mirtazapine confirmation and quantification, liquid-liquid extraction at pH 9–10 was employed on blood and urine samples spiked with deuterated internal standards. GC-MS analysis targeted specific ions (SIM), with quantitative analysis based on the peak area comparison of the analyte to the deuterated internal standard.

Detection of THC involved spiking peripheral blood and urine samples with deuterated internal standards and liquid-liquid extraction at alkaline pH. GC-MS targeted specific ions [SIM] of THC for quantitative assessment.

A separate GC-MS analysis for ethyl alcohol in blood samples was conducted using the “head space” technique. Conditions included an Agilent 7820A equipped with HS Agilent 7694 and an HP-B ALC column. The sample preparation involved the addition of an internal standard (isopropyl alcohol), and quantification was performed with concentrations ranging from 0.25 to 4 g/liter.

In the context of NPS (New Psychoactive Substances) screening, a full scan total ion chromatogram search (m/z 30 to 550 m/z) was conducted, which, however, yielded no results in this case. Targeted searches for all psychoactive substances of pharmacological origin, including those for pain therapy, were performed. GC-FID/headspace for ethanol and volatile compounds were carried out with negative results.

The GC-MS methods were validated and executed using a systematic toxicological analysis methodology, and the identification of active principles was made possible using MassHunter Qualitative Analysis – Unknown software. This software employs advanced data processing and search tools, including integrated deconvolution algorithms, to identify all detectable compounds rapidly and accurately in the samples and easily confirm targets or identify unknown substances. Multiple libraries were concurrently utilized, including NIST2017 and SWGDrug 2019, in our case.

The Limit of Detection (LOD) values for the investigated drugs are summarized in **Table 1**.

**Table 1:** Limit of Detection (LOD) of drugs testing positive.

Representative Compunds	LOD Value (ng/ml)
*Methadone* *EDDP*	5 10
*Mirtazapine*	5
*Tramadol*	5
*Diazepam* *Nordiazepam* *Temazepam* *Oxazepam*	5 5 5 10

### 2.2 Results

In all three cases, the psychiatric history was negative, including substance use disorders. In all three cases, an attempt at resuscitation was performed; furthermore, the cadavers in all three instances exhibited a state of putrefaction ranging between the chromatic and emphysematous phases. From the documentation examined concerning the three deceased individuals, no medical treatment has emerged. All three autopsies exhibited generic findings such as congestion and edema in the parenchymal tissues, particularly in the brain and both lungs, consistent with the intoxication diagnoses and depressant action of the bulbar respiratory center. There were no other macroscopic and/or microscopic features in the corpses that revealed a different mechanism of death.

#### Case #1

Male, age range: 40 to 45 years, weight range: 80 – 85 Kg, height rangeand: 1.65 - 175 m. At the postmortem external inspection, some small abrasions covered by band-aid on the back of the left hand were detected, along with two large bruises on both cubital fossae. Autopsy revealed cerebral oedema (brain weight: 1438 gr.), a globose heart (albeit normal dimensions), and congestion together with pulmonary oedema [left lung weight: 665 gr.; right lung weight: 746 gr). No other features of concern were found. The following samples of biological fluids were taken for the purposes of the toxicology report: central and peripheral blood, urine, stomach content, and bile. The results are shown in **Table 2**.

**Table 2:** Distribution of drugs in subjects as revealed by the toxicologist’s report.

	Central blood	Peripheral blood	Urine	Stomach
Subject #1				
*Mirtazapine (ng/ml)*	76	270	1760	n.d.
*Tramadol (ng/ml)*		49960/ 50970(hydrolyzed)†	584000	n.d.
*Diazepam (ng/ml)*		64	65	n.d.
Subject #2				
*Mirtazapine (ng/ml)*	240	373	3093	n.d.
*Methadone (ng/ml)*	316	649	7800	9980
EDPP* (ng/ml)	208	524	19600	5750
Subject #3				
*Mirtazapine (ng/ml)*		87	820	n.d.
*Methadone (ng/ml)*	370	182	1900	5860
EDPP* (ng/ml)	222	261	700	408
*Diazepam (ng/ml)*		1000	156†	
*Nordiazepam (ng/ml)*		480		
*Temazepam (ng/ml)*		65		
*Oxazepam (ng/ml)*		466		

†Hydrolysed sample. *2-ethylidene-1,5-dimethyl-3,3-diphenylpyrrolidine. n.d., not detected.

In particular, a high tramadol concentration was detected in the biological fluids, in line with the values reported in the scientific literature for deaths caused by acute intoxication (27).

Furthermore, mirtazapine was also detected in toxic ranges.

The histological examination showed acute polyvisceral congestion together with cerebral oedema, which was identified as the death consequent to the consumption of toxics. Moreover, there were no signs of harm or injuries, thus ruling out the homicidal hypothesis.

#### Case #2

Male, age range: 25 to 30 years, weight range: 85 - 95 Kg, height rangeand: 1.65 – 1.75 m. At the postmortem external inspection, there was a sign of acupuncture treatment in the left cubital region, covered with gauze. The autopsy revealed cerebral oedema (brain weight: 1297 gr.) and the lungs were oedematous as well (left lung weight: 633 gr.; right lung weight: 578 gr.). The following samples of biological fluids were taken for the purposes of the toxicology report: central and peripheral blood, urine, and stomach content. The results are shown in **Table 2**.

Methadone concentration values were high and concurred with those found in the literature for deaths caused by acute methadone intoxication.

#### Case #3

Male, age range: 30 to 35 years, weight range: 100 - 110 Kg, height rangeand: 1.75 – 185 m. At the postmortem external inspection there was an abrasion on the right thoracic region, and another on the right wrist. The autopsy revealed cerebral oedema (brain weight: 1498 gr.), while the lungs were both congested and oedematous [left lung weight: 781 gr.; right lung weight: 790 gr.). The following samples of biological fluids were taken for the purposes of the toxicology report: central and peripheral blood, urine, and stomach content. The results are shown in **Table 2**.

After the toxicological examination, the cause of death was attributed to methadone intoxication.

## 3 Discussion

### 3.1 Considerations on the phenomenon of drug abuse in prison conditions

As a part of the phenomenon of drug abuse in prison environments, we need to mention the “new psychoactive substances” (NPS). They refer to a wide variety of different types of drugs with significantly varied effects. The four main types reported in correctional facilities are new synthetic cannabinoids receptor agonists (SCRAs), synthetic cathinones, new benzodiazepines, and new synthetic opioids (28). According to some studies, among the NPS in prison settings, SCRAs have been found to be the predominantly used substances within correctional facilities. These compounds include tert-leucinate indazole carboxamides (e.g., 4F-MDMB-BINACA and MDMB-4en-PINACA), tert-leucinate indole carboxamides (e.g., 5F-MDMB-PICA), and tert-leucinamide indazole carboxamides (e.g., 5F-ADB).

Moreover, according to the European Monitoring Centre for Drugs and Drug Addiction document, it is evident that the four main types reported in prison settings are synthetic cannabinoids, synthetic cathinones, new benzodiazepines, and new synthetic opioids (29).

They demonstrate widespread dissemination within prisons across various parts of the world, with prevalent trafficking occurring through the postal system (30, 31). The use SCRAs in prisons poses organizational and security challenges, with a significant market existing within correctional facilities in many countries (32, 33). Their high potency and ability to be infused into common materials make their detection and control within prisons difficult, while also being responsible for numerous intoxications and deaths (33). Nevertheless, estimating the number of deaths related to NPS in prisons and the general population is complex due to technical and practical challenges, such as the lack of reference standards and low concentrations in biological samples. Deaths related to NPS in prisons are underestimated, with reports in Germany, Latvia, Poland, and the United Kingdom. In England and Wales, between 2013 and 2016, there were 79 deaths related to NPS in prison, of which 56 were self-inflicted, highlighting the potential role of synthetic cannabinoids in exacerbating mental conditions and promoting self-harm (28).

Suicides in prison are more frequently committed by hanging, self-strangulation and self-asphyxiation in general, wrist cutting, and overdose, with varying rates according to the different literature sources (3, 4, 34). However, substance and drug abuse can be a cause of accidental death, along with accidental intoxication of people already taking psychotropic drugs, either for therapeutic or recreational purposes (4, 35). In this regard, the duty of the medical examiner, who must establish whether death was due to homicide, suicide or accident, is extremely challenging.

Prison riots represent a reinforcing mechanism for drug abuse: among self- and non-self-inflicted violent acts, substance consumption is massive and widespread. Documented incidents where drugs have been stolen from the prison infirmary for the purpose of reckless abuse have resulted in mass intoxication and overdose deaths. It could be theorized that prison riots invoke a sort of binge taking of psychotropic substances, but further investigation is needed (17, 25).

In the cases under study, intoxication was due to a combination of substances: tramadol and mirtazapine (Case #1), methadone and mirtazapine (Case #2), and methadone, mirtazapine, and diazepam (Case #3). In Case #1, tramadol concentration values were consistent with findings reported in the literature on acute intoxication, whereas in Case #2 depressant drug concentrations were in the therapeutic range, making it possible to hypothesize synergistic effects in the cerebral nervous system. In this regard, although there are no formally known significant interactions between methadone and mirtazapine, these drugs together can both promote fatal arrhythmias (36–38). On the other hand, Tramadol has been known to show a double toxic effect due to its ability to depress the respiratory function and cause serotonin syndrome, which explains the seizures it can induce; the convulsive effect could be due to monoamine uptake inhibition caused by enantiomers (39–43). In this context, co-ingestion of tramadol and mirtazapine is interesting because of the latter’s noradrenergic and serotonergic effects, which result in synergistic action (21, 23). Cases of acute intoxication due to tramadol and other antidepressant drugs have been documented (11).

In Case #2 and Case #3 the drug that led to death was methadone, which is a synthetic opioid used in the pharmacological treatment of opioid dependence and as heroine replacement in drug addicts [opioid maintenance therapy]. Methadone can lead to death in subjects who take more than their acquired tolerance can cope with [i.e., overdose] and in subjects who use it as a replacement for heroine in drug substitution therapy (44). Furthermore, methadone intake can lead to death even long after ingestion due to gradual intestinal absorption, considering that there is no linear correlation between death and blood concentration (44, 45). Although methadone is considered as reliable, the drug has been shown to affect cardiac functions, especially when associated with other kinds of substances, such as cocaine and benzodiazepines (46–50), resulting in lethal cardiotoxicity. Methadone’s toxic features depress the respiratory function as well as the central nervous system (51). Regarding Case #3, it is noted that benzodiazepines can contribute to death from methadone toxicity by increasing the risk of respiratory depression (52).

In particular, the relationship between methadone and benzodiazepines was confirmed in the cases under study, notably in Case #3. However, the combination of methadone with other drugs makes its role in provoking death more difficult to study.

It is noted that in Case 1 and Case 2, the concentrations of the substance in peripheral blood were higher compared to central blood; the authors deemed it appropriate to consider these data considering available information regarding previous resuscitation attempts, as well as considering the putrefactive state, situated between the emphysematous and chromatic phases, at the time of sample collection (53–55).

Drug abuse in prisons is a chronic issue and prison riots provide the setting for uncontrolled reckless drug abuse since prisoners can gain access to and steal drugs after raiding the infirmary. In the cases reported here, death was caused by substances that are regularly kept in the prison infirmary and regularly administered for the routine treatment of detainees. The high number of detainees needing medical attention and the exceptional nature of the event concurrent with drug intake initially suggested accidental intoxication, yet the intention of the three prisoners cannot be known.

The alternative hypothesis is suicide, but data regarding the hospitalized and deceased inmates’ anamnesis were not accessible, and suicidal ideation was not documented. The binge taking effect of psychotropic substances that we have suggested relies on four hypotheses: (i) drug dependence due to the high prevalence of addicts in prison; (ii) suicidal attempts by non-violent means such as substance ingestion; (iii) accidental overdose of drugs taken for recreational use; (iv) significant reduction in tolerance leading to an increased risk of overdose following prolonged cessation of substance abuse.

### 3.2 Psychiatric considerations

In penitentiary institutions, psychopathological manifestations are particularly prevalent. These may either constitute the continuation or accentuation in prison of pre-existing mental disorders, or conversely, the development of a psychotic response to highly psychotraumatizing events such as imprisonment, remorse for having committed a crime, anticipation of sentencing, or the sentencing itself. Reactions at the psychological level, with their typical phenomenological characteristics, can easily manifest in prison and are facilitated in their development by the prison environment and challenging living conditions. The initial trauma experienced on entering prison is characterized by a range of psychological and often psychosomatic disturbances, and prison entry syndrome occurs more frequently in individuals with higher levels of education, sensitivity, and cultural background (26). Depressive and impulse control disorders are frequently observed in individuals with substance dependence who are subjected to a prison regime (56, 57).

In the three cases studied, precise anamnestic data were not available, but it is certain that the individuals were not undergoing methadone treatment at the time of the event. Subject #2 and Subject #3 took methadone and mirtazapine during the riot (with the addition of benzodiazepines in the case of Subject #3), while Subject #1 took tramadol and mirtazapine. Circumstantial data were not available, and it is unclear whether Subject #1 deliberately chose not to take methadone or whether he arrived when supplies had already been seized and consumed by other inmates. It should be noted, however, that former substance users generally have a good understanding of the effects of methadone and other active substances. In the hypothesis of a history of substance dependence, in cases #2 and #3, it is conceivable that the collective excitement of the rioting crowd exacerbated an impulse control disorder that, as described in the DSM-5, individuals with a history of substance dependence may manifest outside of pathological dependency (not during a withdrawal crisis).

The DSM-5 highlights a crucial aspect of substance use disorders—the enduring alteration of brain circuits, particularly in severe cases. This results in behavioral manifestations such as recurrent relapses and intense cravings triggered by substance-related stimuli. Consequently, ability to exercise volition and self-determination can be compromised, tempting individuals to consume a familiar substance, even in the absence of physical necessity, potentially resulting in therapeutic overdose (26, 58).

Case #1 could have been a former drug addict who failed to obtain methadone during the riot. Alternatively, the individual may not have had a history of drug addiction but was in a fragile state of mind and therefore acted compulsively to mirror the actions of other individuals who were raiding the infirmary at that specific moment. The ‘crowd effect,’ studied by Gustav Le Bon [anthropologist and psychologist] at the late 9th century, is a state of expectant attention in which the individual, ‘immersed’ in a mass of people united by a common purpose, loses inhibitions and succumbs easily to suggestion, adapting their individual actions to the group’s behavior (59, 60).

Such an impulse often manifests due to the fear of not being considered part of the group, coupled with self-underestimation, especially in relation to the prison environment. The hypothesis of suicide suggests a pre-existing depressive disorder in all subjects (DSM-V), which would have made them typically reactive to the detention regime in such a context (26, 61).

In any case, occurrences of this nature, notwithstanding the exceptional nature of the Sars-Cov-2 episode, can be prevented by adjusting the structure and number of beds, implementing mental health treatment programs, training staff, tailoring interventions, and, in general, respecting the rights of inmates and aligning treatments with their conditions. Additionally, in specific cases, residences for individuals with recognized psychiatric disorders may be considered in countries where such facilities exist (62).

### 3.3 Limits

In this instance, psychiatric considerations are provided solely on an observational basis, as there was no prior psychiatric evaluation available before the arrest of the three subjects, nor an ongoing psychiatric assessment during detention. This constitutes the primary limitation. Furthermore, we have only described a limited case series; for a comparison with the international literature, it would be necessary to have a larger sample size with complete anamnestic data available.

Ultimately, the toxicological examination on the keratinic matrix could not be conducted in the present instance. It is represented, however, that although not carried out in this case, the investigation on keratinized matrix can prove useful for further insight into past consumption (63).

Regarding the limits related to toxicological examinations, it should be noted that the analysis technique employed, GC-MS, is inherently inadequate for identifying several low-dose active substances, including NPS, despite conducting a full scan total ion chromatogram search. In this regard, it is important to recall that the cases were studied comprehensively, also relying on the available circumstantial data.

## 4 Conclusion

Prison riots are correlated to a high number of deaths and injuries as a result of drug overdose after stealing from the prison infirmary. Raids are usually carried out by drug addicts or individuals with suicidal intentions. The phenomenon is complex and challenging to study, but it is particularly necessary to focus on the prevention of raids in the first place.

## Data Availability

The original contributions presented in the study are included in the article/supplementary material, further inquiries can be directed to LT, luca.tomassini@unicam.it.
